# Eight-year-old Boy with New-onset Seizure

**DOI:** 10.5811/cpcem.2019.4.42547

**Published:** 2019-04-12

**Authors:** Lauren Rosenblatt, Danya Khoujah, Zachary D.W. Dezman, Laura J. Bontempo

**Affiliations:** * University of Maryland Medical Center, Department of Emergency Medicine, Baltimore, Maryland; † University of Maryland School of Medicine, Department of Emergency Medicine, Baltimore, Maryland

## Abstract

An eight-year-old boy presented to the emergency department for a first-time seizure. The patient had only signs of mild dehydration on physical exam and had an uneventful postictal recovery. First-time seizures in pediatric patients are often benign and require only an outpatient workup; some are dangerous. This case takes the reader through the differential diagnosis and systematic work-up of new-onset pediatric seizures, leading to an unanticipated diagnosis.

## CASE PRESENTATION

An eight-year-old African-American male was brought to the emergency department (ED) by ambulance after a first-time, witnessed seizure at home. The patient arrived approximately 15 minutes after the seizure and was somnolent but arousable and confused, consistent with a postictal state. The remainder of the history was taken from the patient’s mother, who was at his bedside. She stated that the patient had been feeling unwell for the past two to three days. He had been complaining of upper respiratory infection symptoms, including cough and nasal congestion. The mother stated she heard a “thud” upstairs and ran up to find her son on the floor shaking and incontinent of his bladder and bowels. The shaking lasted about one to two minutes. She reported that her son had a decreased appetite recently, but even when well he was a very picky eater with a very limited diet. He had only eaten French fries since becoming sick. The patient’s mother had been encouraging oral hydration with Gatorade and Pedialyte.

The patient had no significant past medical history. He had never had surgery or other hospitalizations. He had an allergy to amoxicillin which resulted in hives. He saw a pediatrician regularly and was up to date on vaccines. He did not take medications on a daily basis. There was a family history of hypertension but no family history of seizure disorders. They lived in a home, and mother stated they felt safe at home. No one else in the home had been ill.

Vital signs included an oral temperature of 38° Celsius, a heart rate of 108 beats per minute, blood pressure of 111/70 millimeters of mercury, breathing at a rate of 26 breaths per minute, and oxygenating 97% on room air. On initial physical exam, patient appeared drowsy, but overall was well developed, with a body mass index of 19 kg/m^2^, and in no acute distress. His head was normocepahlic and atraumatic. Extraocular movements were intact, pupils were three millimeters and equal, round, and reactive to light. His tympanic membranes were normal and nose was normal. He had dry mucous membranes and his oropharynx was clear without exudate or erythema. His neck was supple and without lymphadenopathy. His lungs were clear to auscultation bilaterally, with good air movement. There were no wheezes, rales, or rhonchi. Auscultation of the patient’s heart revealed a normal rate and regular rhythm without murmurs, rubs, or gallops. Abdomen was soft, non-tender, and non-distended with normal bowel sounds. The extremities had no edema, 2+ distal pulses, no tenderness, and no deformity with normal range of motion. Neurological exam revealed that he was easily arousable without cranial nerve deficits, normal strength, normal sensation, normal coordination, and normal gait. His skin was warm and dry, without rashes, pallor, or jaundice. He had a capillary refill of three to five seconds in all extremities.

After approximately 30 minutes in the ED, the patient’s mother reported that her son seemed to be more alert, interactive, and conversive, and was back to his baseline mentation.

**Image 1:**
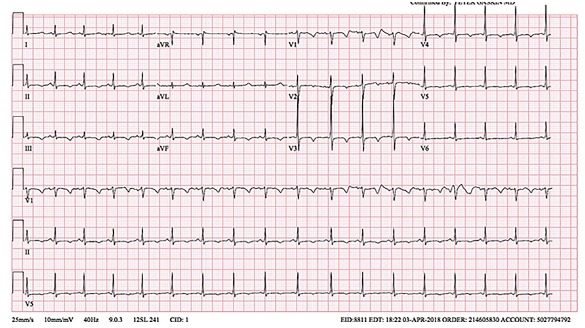
Electrocardiogram of eight-year-old boy with new-onset seizure.

**Image 2:**
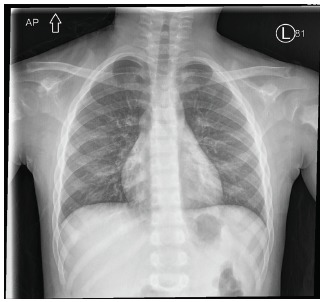
Chest radiograph of eight-year-old boy with new-onset seizure.

Labs, electrocardiogram (ECG), and chest radiograph were obtained in the ED. Laboratory results are shown in the table. ECG and chest radiograph can be seen in Images 1 and 2, respectively.

While in the ED, a test was ordered, and a diagnosis was made.

## CASE DISCUSSION

When I first read this case, it was unclear to me why it was a diagnostic dilemma. This is a case of an eight-year-old boy presenting with a seizure. What am I missing? There must be more to this than meets the eye.

He presented after a witnessed seizure at home, accompanied by incontinence, and followed by a postictal period. The seizure was preceded by upper respiratory infection symptoms and decreased oral intake. His physical exam is significant for signs of dehydration, such as tachycardia, dry mucous membranes, delayed capillary refill and tachypnea. His neurological exam is consistent with the described postictal period (drowsy and easily arousable), with intact cranial nerves, power, and sensation. He returned to his baseline mental status within approximately 30 minutes of arrival, which is what would be expected.

While all these descriptors of the incident make it typical for a first-time seizure, an alternative explanation for “seizure-like” activity should also be considered. Most importantly, the clinician must consider arrhythmias. The ECG provided reveals sinus tachycardia with no evidence of arrhythmia or an arrhythmogenic disorder such as hypertrophic cardiomyopathy or Wolff-Parkinson-White syndrome. Arrhythmia, therefore, is less likely to be the etiology.

In an attempt to understand what about this seizure makes it different, I went through my differential diagnosis for provoked seizures:

- Structural lesions
- Trauma
- Vascular
- Infection
- Toxic
- Metabolic
- Hypertensive
- Heat stroke
- Pregnancy (eclampsia)

The last three entities on this list (hypertensive encephalopathy, heat stroke and pregnancy) can be excluded right away, given the normal blood pressure, temperature and male gender.

A structural lesion, such as a tumor, is unlikely in the absence of signs of increased intracranial pressure or a focal neurological deficit. Less emergent lesions, such as congenital anomalies, can be safely addressed with an outpatient magnetic resonance imaging (MRI), excluding this entity from the differential. Trauma is unlikely to be the cause, as there is no reported trauma and no signs of head trauma on the patient’s examination. A vascular catastrophe, such as an intracranial hemorrhage, would be accompanied by a headache, vomiting, and/or neurological findings, all of which are absent in this case. This, too, is therefore excluded. The remaining possibilities of infectious, toxic and metabolic causes of the seizure must be looked at more closely.

### Infectious

Here I have to consider meningitis/encephalitis, febrile seizures and a brain abscess. Although the patient has infectious symptoms, he does not have features suggestive of a more serious infection, such as neck stiffness, headache, vomiting, rash, or persistent altered mental status (AMS). I cannot exclude meningitis/encephalitis, but I will place them low on my differential diagnosis. Febrile seizures are excluded by the lack of fever on presentation as well as the child’s age, which is outside the typical range for febrile seizures (three months to three years). An intracranial abscess could be considered if the patient were immunocompromised or had fever or focal neurological deficit. The patient is afebrile without focal neurological deficit and, as far as is known, is immunocompetent. An intracranial abscess is therefore excluded. The only diagnosis that remains from this category is meningitis/encephalitis.

### Toxic

Here I have to consider several agents that can cause seizures, whether accidentally administered in large doses by the parent, or accidentally (or intentionally) ingested by the child. Many over-the-counter (OTC) cold medications contain anticholinergics, which in toxic quantities can cause seizures. Although some elements of the current presentation are consistent with an anticholinergic overdose, such as the AMS, tachycardia and dry mucous membranes, others are not, including the patient’s pupillary exam. The patient’s mother did not report any OTC medication use; however, children can surreptitiously ingest medications that are present at home and were inadvertently omitted from the initial history-taking, leaving anticholinergic overdose as a tempting explanation for the seizure. Other toxins that induce seizures include lithium, isoniazid and tricyclic antidepressants, which the child is not reported to have access to. Furthermore, there were no signs of tricyclic toxicity on the ECG, such as a rightward axis, wide QRS or terminal R wave in aVR. Finally, alcohol and benzodiazepine withdrawal can cause seizures, although these would present along with tachycardia, tremors, hypertension, diaphoresis, tongue fasciculations, and AMS. Outside of the mild tachycardia, none of these other signs are present. Neither is there past medical or social history suggesting regular ingestion of benzodiazepines or alcohol, effectively excluding these culprits as well. Other than anticholinergic overdose, toxin-induced seizures are off my list.

### Metabolic

Several metabolic derangements can lead to seizures, including sodium abnormalities (hyper- or hyponatremia), glucose abnormalities (hyper- or hypoglycemia), hypocalcemia, hypomagnesemia, or every emergency physician’s least favorite diagnosis to navigate in a child: inborn error of metabolism (IEM). The answer may be as simple as obtaining an electrolyte panel, which should include magnesium and calcium. As for IEM, the child’s age and lack of other medical history makes it much less likely. Combined with a lack of acidosis as evidenced by a normal serum bicarbonate level (21 millimoles per liter [mmol/L]) and normal lactate (0.7 mmol/L), and a normal glucose level, IEM is virtually excluded. Serum levels of sodium, glucose, and magnesium are all within normal limits. The calcium level, on the other hand, is 5.1 milligrams per deciliter (mg/dL), with a normal range of 8.5 – 10.2 mg/dL. Could it really be this low, or is it a lab error?

Additionally, I noted that the patient did not receive any neuroimaging. Although it is tempting to think that neuroimaging would reveal the etiology of his seizures, in truth, very rarely does neuroimaging of a pediatric patient with a first-time, non-febrile seizure affect the management of the patient.^1^ My differential diagnosis is, therefore, unaffected by the absence of a computed tomography.

In review, my working differential diagnosis includes meningitis/encephalitis, anticholinergic overdose, and hypocalcemia. Taking another look at the ECG, the QTc is prolonged, which is consistent with hypocalcemia or an anticholinergic overdose. The patient’s serum albumin level is 3.9 grams per deciliter, which is normal. Using the formula – corrected calcium = [0.8 x (normal albumin – patient’s albumin)] + serum calcium level – the patient’s corrected calcium level is 5.2 mg/dL, which is still very low. The corrected calcium level and the patient’s ECG suggest that his hypocalcemia is likely the culprit for the seizure. In addition, given that the question that ends the case is “a test was ordered, and a diagnosis was made,” and knowing that anticholinergic levels are not an accessible test from the ED, the diagnosis of anticholinergic overdose is excluded. I believe the diagnostic test of choice in this patient case is an ionized calcium level. However, what was the primary cause of the patient’s hypocalcemia?

Hypocalcemia in children can be caused by hypoparathyroidism, low vitamin D levels (due to intake or synthesis), as a side effect of a medication/toxin, or secondary to another abnormality (alkalosis or hypomagnesemia) or a severe illness (such as pancreatitis or sepsis). The last three entities can be easily excluded, as the child is not on any daily medications, his serum bicarbonate and magnesium are normal, and he does not appear toxic. Hypoparathyroidism can be due to a genetic disorder or secondary to a thyroid surgery. It is unlikely that this is a first presentation of a genetic disorder at the age of eight, and the child has not had any surgeries. This leaves low vitamin D levels as a possible culprit for the hypocalcemia, whether due to decreased dietary intake, decreased absorption, or inadequate exposure to sunlight. Looking back over the case details, the mother did mention that the patient is a picky eater, making a dietary cause for the hypocalcemia most likely.

## CASE OUTCOME

**Image 3:**
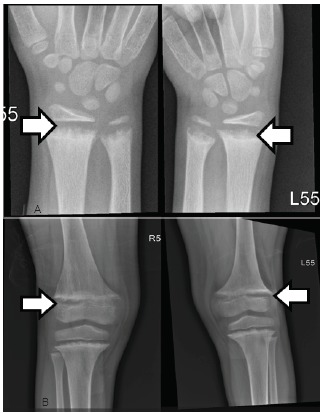
Radiograph of bilateral wrists (A) and knees (B) of an eight-year-old boy with new-onset seizure. Arrows denote areas of poor bone mineralization.

The first thought once the labs started to result was that the calcium value must be wrong. A repeat calcium and an ionized calcium level were obtained, which were 5.0 mg/dL (range 8.8–10.8 mg/dL) and 0.77 mg/dL (range 1.15–1.29 mmol/L). This confirmed hypocalcemia without any other obvious electrolyte abnormalities, concluding that the patient had suffered a hypocalcemic seizure. This is consistent with the ECG, which showed QTc prolongation of 456 milliseconds, a common complication of hypocalcemia. The patient was given intravenous calcium gluconate in the ED. Endocrinology was consulted and they recommended obtaining radiographs of the wrists and knees (Image 3). These showed degradation and demineralization at the distal metaphases, revealing the diagnosis of rickets. Further investigation and conversation with the patient’s mother regarding her child’s picky eating habits revealed that her son only eats chicken nuggets and French fries, even when feeling well.

The patient was admitted to the pediatric hospitalist team. Inpatient workup included obtaining a parathyroid hormone (PTH) level and vitamin D studies. The results of those studies were vitamin D 25-hydroxy of <4 nanograms (ng)/mL) (range 20–80 ng/mL), vitamin D 1,25-hydroxy of 10.5 ng/mL (range 19.9–79.3 ng/mL), and parathyroid hormone (PTH) of 636 picograms (pg)/mL) (range 8–54 pg/mL), proving that the etiology of the rickets was secondary to a vitamin D nutritional deficiency. The patient was started on aggressive calcium and vitamin D replacement. At six-month follow-up, repeat laboratory studies showed some improvement with a calcium of 8.5 mg/dL; however, vitamin D 25 hydroxy was still low at 4.2ng/mL.

## RESIDENT DISCUSSION

Rickets is defined as a disease resulting from the failure to mineralize newly formed osteoid at the growth plates of long bones, causing an abundance of unossified cartilage.^2^,^3^ Rickets is specifically a pediatric disease and can only occur prior to fusion of the epiphyses of bones. After this time, the demineralization of bone results in osteomalacia in adults.

The presence of rickets in the literature has been documented as early as the 17^th^ century by scientists Whistler and Glisson.^2^,^4^ Since then, there has been an abundance of literature documenting the role of vitamin D in the absorption and utilization of calcium through various organ systems within the human body. Public health efforts across the globe have included fortifying food with vitamin D; however, rickets remains prevalent, even in industrialized countries. The Centers for Disease Control and Prevention estimates that approximately five in 1,000,000 children between the ages of six months and five years in the U.S. has rickets.^3^ Rickets can be caused by renal, gastrointestinal, hepatic, or metabolic disease that prevent vitamin D absorption and metabolism. However, nutritional vitamin D deficiency is the leading cause of rickets globally.

The clinical symptoms of rickets tend to present in a bimodal fashion, with the majority of cases presenting during infancy, followed by a small resurgence during adolescence. This is thought to be associated with time periods of high bone turnover during normal growth and puberty.^2^ During infancy, nutritional rickets is primarily caused by exclusive breastfeeding without formula or vitamin supplementation. It is thought that the increase in rickets cases documented in the U.S. is most likely secondary to the increase in popularity of breastfeeding over formula feeding. Breast milk only contains approximately 15–50 international units (IU)/L of vitamin D, which is well below the daily recommended requirement of 200–600 IU/L.^5^,^6^

During adolescence, the cause of nutritional rickets is primarily the combination of poor sun exposure and dark pigmented skin. An overwhelming number of articles highlight that the majority of documented cases of rickets occurs in Black and dark-skinned children. Review articles reporting on the prevalence of rickets in the U.S. and Canada consistently report that 83% to 91% of cases are in Black or dark-skinned patients.^5^,^7^,^8^

Vitamin D plays an integral role in the regulation of intracellular and extracellular calcium concentrations within the human body. Vitamin D is either absorbed through photochemical synthesis of sunlight and ultraviolet light through the skin or by dietary consumption. Specifically, vitamin D_3_ (cholecalciferol) is absorbed in the skin by 7-dehydrocholesterol. However, in darkly pigmented children, melanin competes with 7-dehydrocholesterol resulting in poor uptake of cholecalciferol.^3^,^7^ Vitamin D_2_ is consumed by diet. Both forms are ultimately converted by the liver and kidney into the functional form of 1,25-dihydroxyvitamin D (calcitriol). Calcitriol functions with PTH to help balance concentrations of calcium in the serum. Calcitriol stimulates absorption of calcium by the gut or mobilizes calcium and phosphorus from the bone to maintain normal serum calcium levels.

Rickets has been classically defined by the obvious physical exam findings of frontal bossing (protrusion and widening of the forehead), bowed long bones, short stature, and rachitic rosary (beading of the costochondral junctions of the ribs). These, however, are late signs of rickets and uncommonly seen in the U.S or other developed countries. The literature more commonly documents cases with new-onset seizure, severe muscle spasms and tetany, cardiac arrhythmias, and new bone fractures.^6^,^7^,^9^ ED work-up for these presentations may reveal low serum concentration of calcium; however, diagnosis of rickets is ultimately made by radiographs of long bones showing cupping, fraying and demineralization at the metaphysis.^2^ The etiology of the rickets requires further work up. Nutritional rickets is specifically diagnosed from lab work showing low serum levels of 25-hydroxyvitamin D (calcidiol)^7^. The patient described in this case was noted to have a calcidiol level of <4 ng/mL (range 20–80 ng/mL), confirming vitamin D-deficient nutritional rickets.

Treatment includes giving intravenous (IV) calcium gluconate for patients presenting with symptomatic hypocalcemia, including seizures, or those with notable ECG changes. For those who present with new-onset seizure or hypocalcemia seizures, there is no evidence for using antiepileptic drugs for seizure prevention. Most articles discuss treatment of the underlying condition and providing vitamin D and calcium supplementation. IV calcium gluconate should be dosed at 100–200 mg/kg/dose with a maximum dose of 1–2 grams. Aggressive oral supplementation should be initiated as well. Vitamin D treatment should be 50,000 IU/L weekly or 2000 IU daily, with a calcium-rich diet.^6^,^9^

## FINAL DIAGNOSIS

Nutritional vitamin D deficiency (rickets).

## KEY TEACHING POINTS

1. Hypocalcemic seizures, tetany, and cardiac arrhythmias are common and concerning ED presentations of rickets.
2. Initial treatment, if presenting with concerning symptoms, is IV calcium gluconate.
3. Diagnosis can be made in the ED by radiographs of the long bones.

## Figures and Tables

**Table:** Laboratory results for an eight-year-old boy with a new-onset seizure.

Labratory Test	Value
Complete blood cell count	
White blood cells	5.3 K/mcl
Hemoglobin	12.9 g/dL
Hematocrit	39.3%
Platelets	199 K/mcl
Serum chemistry	
Sodium	142 mmol/L
Potassium	3.9 mmol/L
Chloride	106 mmol/L
Bicarbonate	21 mmol/L
Blood urea nitrogen	4 mg/dL
Creatinine	0.33 mg/dL
Glucose	95 mg/dL
Magnesium	1.6 mg/dL
Phosphorus	3.9 mg/dL
Calcium	5.1 mg/dL
Albumin	3.9 g/dL
Lactate	0.7 mmol/L
Thyroid stimulating hormone	0.26 mIU/L

*K/mcl*, thousands per microliter; *g/dL*, grams per deciliter; *mmol/L*, millimoles per liter; *mg/dL*, milligrams per deciliter; *mIU/L*, milli-international units per liter.
